# An efficient method for miRNA detection and localization in crop plants

**DOI:** 10.3389/fpls.2015.00099

**Published:** 2015-03-03

**Authors:** Flor de Fátima Rosas-Cárdenas, Rocío Escobar-Guzmán, Andrés Cruz-Hernández, Nayelli Marsch-Martínez, Stefan de Folter

**Affiliations:** ^1^Laboratorio Nacional de Genómica para la Biodiversidad, Centro de Investigación y de Estudios Avanzados del Instituto Politécnico NacionalGuanajuato, México; ^2^Centro de Investigación en Biotecnología Aplicada del Instituto Politécnico NacionalTlaxcala, México; ^3^Facultad de Ingeniería, Universidad Autónoma de QuerétaroQuerétaro, México; ^4^Departamento de Biotecnología y Bioquímica, Centro de Investigación y de Estudios Avanzados del Instituto Politécnico NacionalGuanajuato, México

**Keywords:** tissue-printing, miRNA detection, hybridization, fruits, miR164

## Abstract

microRNAs are a class of non-coding small RNAs (sRNAs) that are important regulators of gene expression at the post-transcriptional level by mRNA cleavage or translation inhibition. Another class of sRNAs are siRNAs, which also regulate gene expression but by causing DNA methylation. This epigenetic regulatory role has been observed for some miRNAs as well. The use of sRNAs allows the development of biotechnological applications in plants. To develop these types of applications, and to better understand the natural roles they play, it is important to be able to detect and to localize these sRNAs at the plant tissue level. Sometimes, in crop plants this can be challenging. Therefore, we developed a tissue printing hybridization protocol for easy and efficient detection of sRNAs and demonstrate this by the analysis of the spatio-temporal expression patterns of the miRNAs miR159 and miR164 in fruits of various crop plants. Moreover, we show the possibility to also detect the expression of miRNAs in fruit juice using a dot blot hybridization approach.

## INTRODUCTION

microRNAs (miRNAs) are a class of small RNAs (sRNAs) that are fundamental regulatory elements of eukaryotic genomes (Voinnet, 2009) and their widespread conservation and divergence in the plant kingdom has been demonstrated (Chavez Montes et al., 2014). Detection of the spatio-temporal expression of miRNAs is critical to understand their function (van Rooij, 2011). Several techniques such as Northern-blot hybridization (Pall and Hamilton, 2008), microarray analysis (Yin et al., 2008), stem-loop RT-PCR analysis (Chen et al., 2005), or sRNA-seq analysis (Chavez Montes et al., 2014) permit the identification of miRNA expression patterns, which may suggest their involvement in certain biological processes. Besides these advantages, a drawback of these techniques is the lack of information about the actual localization of the miRNA in the tissue or organ itself, which is important for understanding the biological function of the miRNA.

A technique overcoming this drawback is *in situ* hybridization, which also allows the detection of miRNAs in thin tissue sections using a labeled complementary probe against the miRNA of interest (Kidner and Timmermans, 2006; Várallyay and Havelda, 2011). Nevertheless, the generation of thin tissue sections from large tissues using paraffin-embedding or cryosections may be challenging. Tissue printing in nitrocellulose or nylon membranes is a technique employed to study the localization of proteins, nucleic acids, and soluble metabolites from freshly cut tissue slices. Tissue printing has been defined as ‘the art and science of visualizing cellular material and information that are transferred to a receptive surface when the cut surfaces of section of tissues or organs are pressed against such a surface’ (Varner and Ye, 1994). This technique does not require RNA extraction or the preparation of thin tissue sections, and it allows the simultaneous analysis of many samples. Therefore, this technique is especially convenient for large tissues or organs, such as fleshy fruits that are often difficult to section for *in situ* hybridizations. Tissue printing in combination with hybridization has been used successfully to determine mRNA and protein localization in several studies (Holland et al., 2000; Qu et al., 2003; Jolie et al., 2010; Pluskota et al., 2011; Esteves et al., 2013; Mochizuki and Ohki, 2015). However, to date almost no examples have been reported for the detection of miRNAs in plants using tissue printing (e.g., Rosas-Cárdenas et al., 2015).

Here we report a protocol for tissue printing combined with hybridization in order to detect and to localize known miRNAs at the tissue level in different species. Although we focused mainly on fruits, this protocol may be used for other fleshy tissues as we demonstrate here by detecting miRNAs in floral buds. Moreover, we show also the possibility to detect the expression of miRNAs in fruit juice using a dot blot hybridization approach.

## MATERIALS AND METHODS

### PLANT MATERIAL

Fleshy fruits were purchased at the local market. Agave buds (*Agave atrovirens*) were collected at the Centro de Investigación y de Estudios Avanzados del Instituto Politécnico Nacional (CINVESTAV-IPN), Irapuato. The tissues were washed and dried at room temperature.

### REAGENTS

Nylon membrane (Hybond NX; Amersham/Pharmacia, cat. no. RPN303T)
3MM Whatman® chromatography paper
1-Methylimidazole (Sigma–Aldrich)
1-ethyl-3-(3-dimethylaminopropyl) carbodiimide (EDC; Sigma–Aldrich)
Rapid-hyb Buffer (GE Healthcare)
EasyTides® Adenosine 5′-triphosphate, (γ-^32^P)-6000 Ci/mmol, 10 mCi/ml (370 mBq/ml), 50 mM Tricine (pH 7.6) (Perkin Elmer)

### BUFFERS AND SOLUTIONS FOR HYBRIDIZATION ANALYSIS

• EDC fixation solution (24 ml)
245 μl of 12.5 M methylimidazole, pH 8.0
0.5 g EDC
• Wash solution
2x SSC
0.1% SDS

### TISSUE PRINT

The samples were washed and dried at room temperature. The samples were cut in longitudinal and/or transverse sections. After cutting the samples, they were immediately placed with the cut surface face down on the membrane (Amersham Hybond-N; GE Healthcare). The different sections were firmly pressed on the nylon membrane for 30 s, subsequently the tissue was carefully removed and the membrane was dried (around 5–20 min) at room temperature.

### FIXATION OF THE MEMBRANE

The EDC fixation solution was prepared as describe Pall and Hamilton (2008) with some modifications. Briefly, 0.753 g of EDC was dissolved in 10 ml of water, 245 μl of 12.5 M 1-methylimidazole was added and finally 150 μl of 1 M HCl was added to obtain a pH of 8. This solution was prepared fresh before use. The membranes were incubated in this fixation solution for 1 h at 65°C, and then rinsed twice with water. The membranes were dried at room temperature, and stored at -20°C till further use. miRNA detection was carried out as the hybridization analysis.

### HYBRIDIZATION ANALYSIS

To prepare the probes we used the following synthesized oligonucleotides, which sequences are complementary to each 21 nucleotide mature miRNA of interest: 5′-AGGGGCCATGCTAATCTTCTC-3′, 5′-AAGAGCT CCCTTCAATCCAAA-3′, 5′-UGGAGAAGCAGGGCACGUGCA-3′, and 5′-TGCACGTGCCCTGCTTCTCCA-3′, to detect the small nucleolar RNA U6 (positive control), miR159a, miR164, and miR164* sense (negative control), respectively. The small nucleolar RNA U6 is often used as a loading control or used for signal normalization (e.g., Kou et al., 2012; Rosas-Cárdenas et al., 2015). To prepare the probes the oligonucleotides were labeled as follows: 4 μl of oligonucleotide 100 μM, 1 μl of T4 Kinase (10 U/μl), 1 μl [γ-^32^P] ATP (10 mCi/ml), 4 μl of forward buffer and 10 μl of water; the reaction solutions were incubated at 37°C for 1 h and subsequently added to the membranes. The hybridization was made as we described before in Rosas-Cárdenas et al. (2011) with some modifications. In summary, the used hybridization solution was the Rapid-hyb buffer (GE healthcare), which contains chemical blocking agents and therefore does not require heterologous DNA to control non-specific binding of probes to the membrane. The membranes were pre-hybridized with 15 ml hybridization solution for 1 h at 42°C with constant agitation, followed by adding the labeled probe of interest, and then incubated for 24 h at 42°C with constant agitation. Membranes were washed with wash solution (2x SSC, 0.1% SDS), first for 4 min, and afterwards for 2 min at room temperature, followed by exposure to a storage phosphor screen for 24 h and/or 48 h at room temperature (highly expressed miRNAs can be detected already at 12 h). Finally, the storage phosphor screen was scanned in a Storm 860 Gel and Blot Imaging System (Amersham Biosciences). This protocol is not yet tested with the use of non-radioactive probes, but we expect it to work.

### SUMMARY: TISSUE PRINTING HYBRIDIZATION PROTOCOL

1. Cut a membrane to the appropriate size.
2. Cut the tissue and carefully place a longitudinal and/or a transverse section(s) onto the membrane with the cut surface down and firmly press the tissue for 30 s.
3. Carefully remove the tissue from the membrane.
4. Dry the membrane at room temperature.
5. Fix the membrane with EDC solution for 1 h at 65°C.
6. Rinse the membrane with water and then dry it at room temperature.
7. Pre-hybridize the membrane with 15 ml hybridization solution for 1 h at 42°C with constant agitation.
8. Add the labeled probe of interest and incubate for 3–24 h at 42°C with constant agitation.
9. Discard the hybridization solution and wash the membrane with wash solution for 4 min at room temperature.
10. Discard the wash solution and wash the membrane again for 2 min at room temperature.
11. Expose the membrane to a storage phosphor screen for 24 h at room temperature and then scan the screen and analyze the signal.

## RESULTS AND DISCUSSION

**FIGURE 1:**
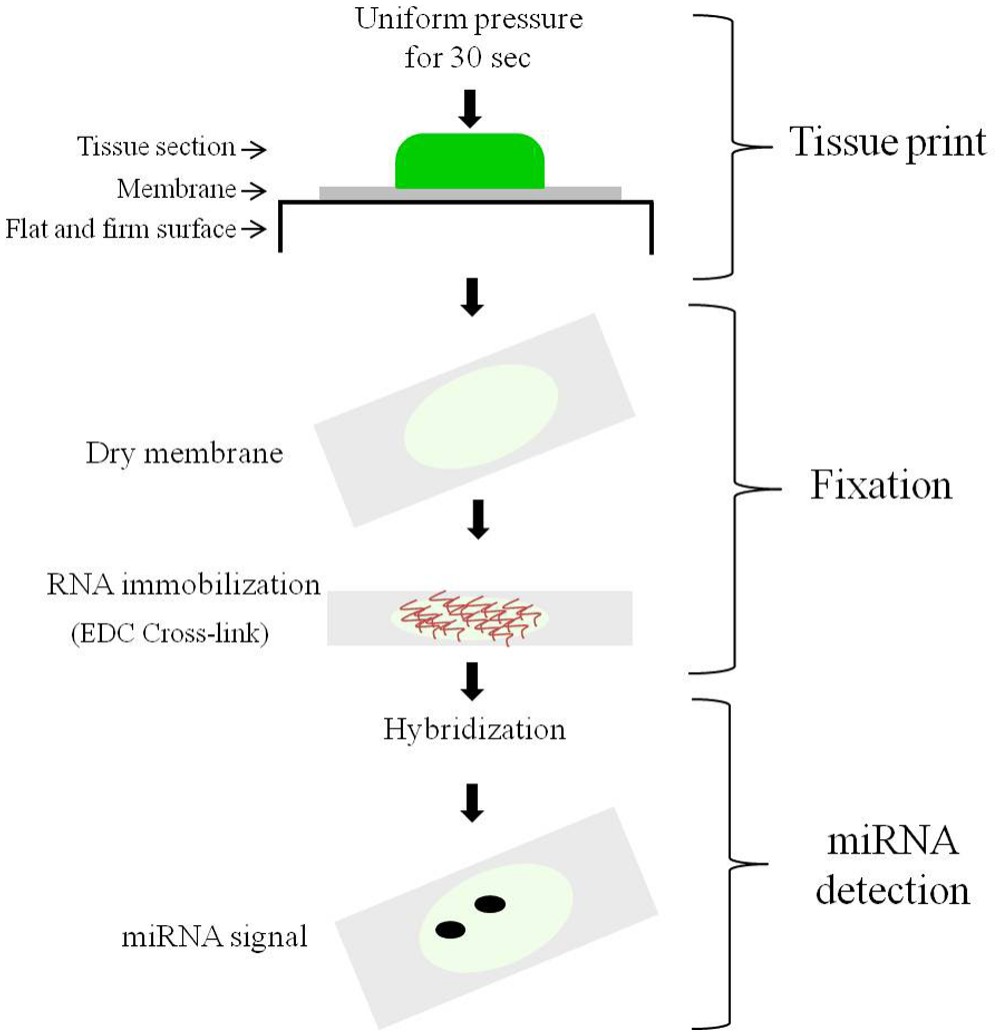
**The procedure of the tissue printing hybridization protocol.** The diagram illustrates the main steps: (1) The tissue printing step; (2) the membrane fixation step; and (3) the hybridization detection step.

**FIGURE 2:**
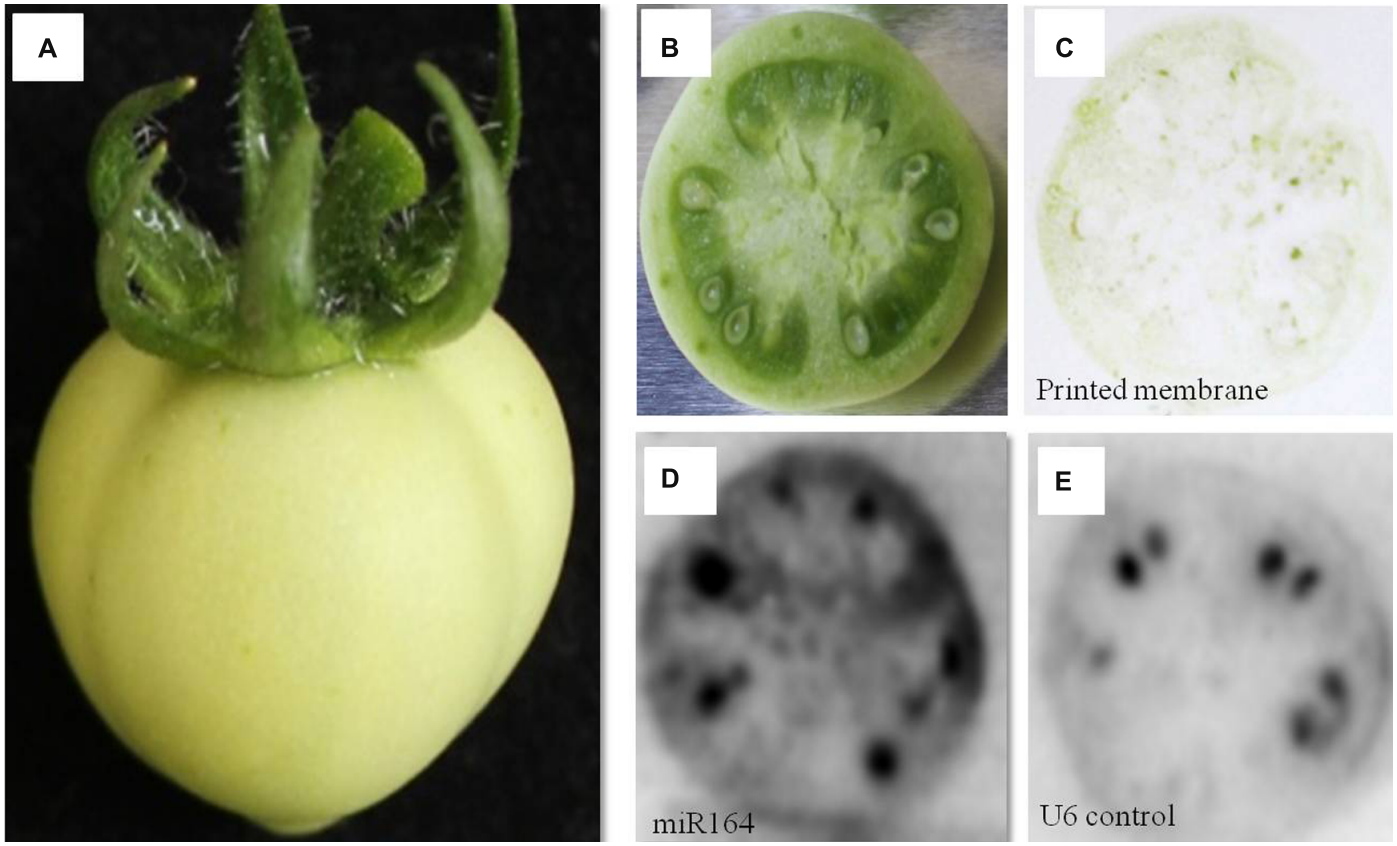
**miR164 detection by tissue printing hybridization in tomato fruit. (A)** Tomato fruit; **(B)** Transverse cut tomato fruit to be used for the tissue printing; **(C)** Tissue print on the membrane made from a transverse cut tomato fruit; **(D)** miR164 detection by hybridization; **(E)** nucleolar U6 (positive control) detection by hybridization.

In this work we aimed to develop an easy protocol that would allow the *in situ* detection of miRNAs in relatively large plant tissues or organs. For this we used the combination of tissue printing and hybridization. Tissue printing is a simple procedure by which the uppermost layer of cells and the surrounding extracellular matrix of the cut surface from plant tissue are transferred to a membrane after physical contact with the membrane surface generating a bidimensional anatomical image on which the molecule of interest can be visualized by hybridization. The procedure is depicted in **Figure 1** as a flow diagram consisting of three steps: tissue print, membrane fixation, and miRNA detection. The detailed procedure is described in Section “Materials and Methods.” We used this tissue printing hybridization protocol to observe the spatio-temporal expression of miR159 and miR164 in different tissues (**Figures 2**–**Figure 4** Supplementary Figure S1), as described below.

**FIGURE 3:**
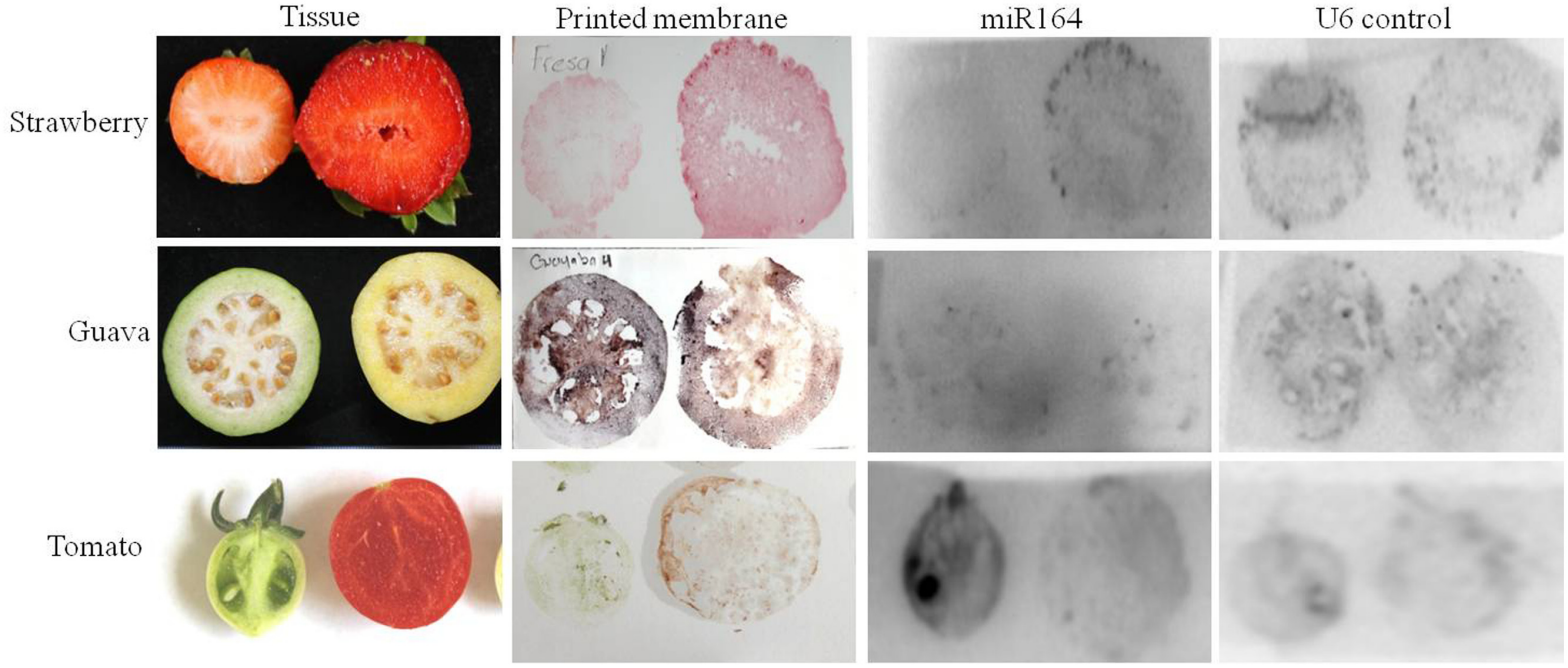
**miR164 detection by tissue printing hybridization in different plant species.** Fruits (strawberry, guava, and tomato) were cut in half and then printed on the membrane. The membranes were fixed and then hybridized with ^32^P-labeled oligonucleotide probe complementary to miR164 or to the nucleolar U6 (positive control).

**FIGURE 4:**
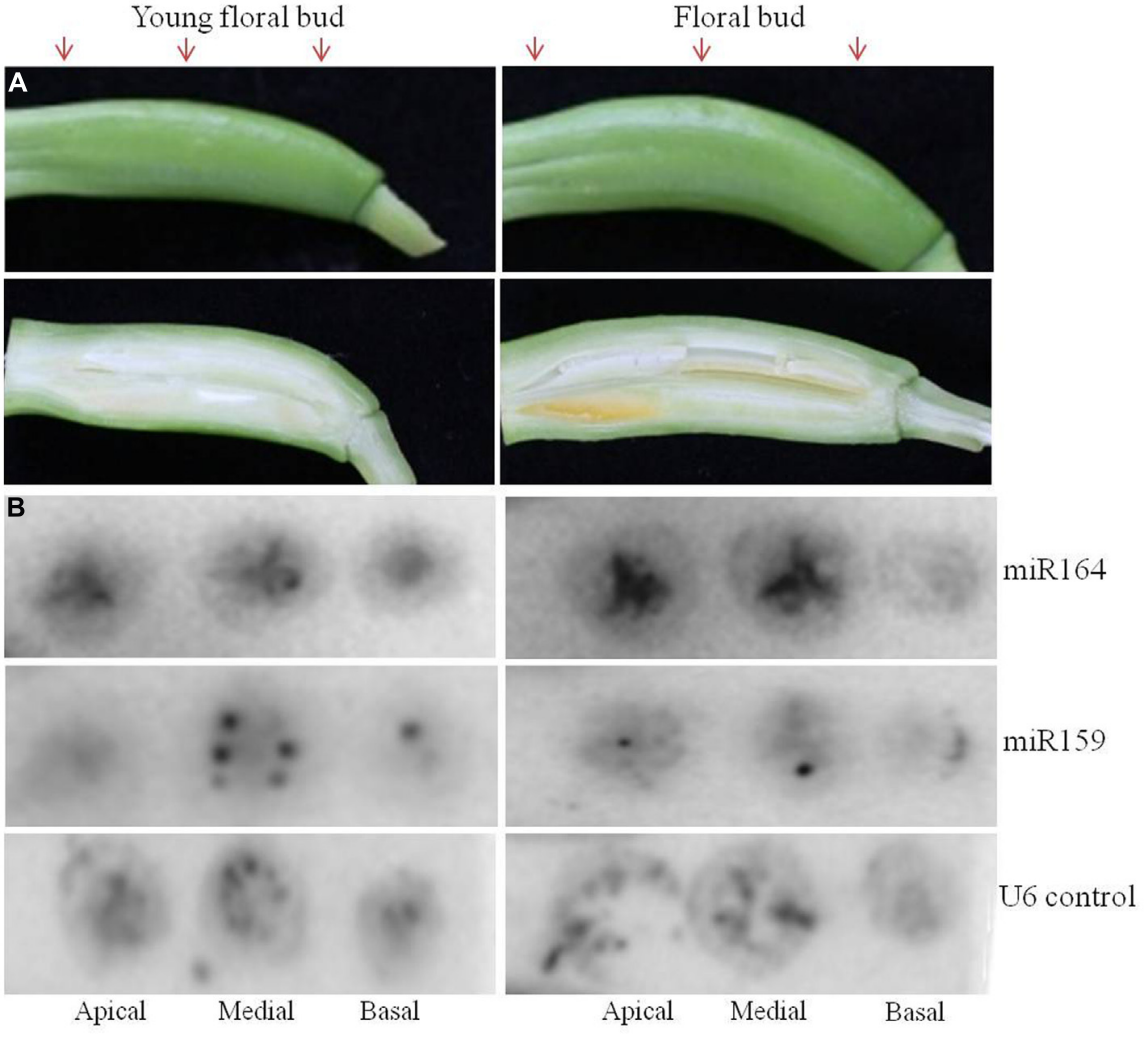
**Spatio-temporal expression of miR164 and miR159 by tissue printing hybridization in floral buds of agave.** Expression of miR164 and miR159 was analyzed in the apical, medial, and basal region of agave floral buds by tissue printing hybridization. Two different stages of agave floral buds were analyzed. **(A)** Transverse sections of the agave floral buds were used to make the tissue prints; arrows indicate the apical, medial, and basal region were the cuts were made. Below each floral bud a longitudinal section is shown to indicate the developmental stage. **(B)** Detection of miR164, miR159, and nucleolar U6 (positive control) in agave floral buds by hybridization.

### DETECTION OF miRNAs BY TISSUE PRINTING HYBRIDIZATION

We first tried the protocol with a green tomato fruit (**Figure 2A**). A transverse cut of the tomato fruit was made (**Figure 2B**), which was then firmly pressed on the membrane, leaving a faint tissue print behind on the membrane (**Figure 2C**). The membrane was then hybridized with a ^32^P labeled probe against miR164 (**Figure 2D**) and an independently printed membrane hybridized with a probe against the nucleolar U6 (positive control) (**Figure 2E**), and with a miR164 sense probe (negative control; Supplementary Figure S2). A strong miR164 signal was observed in seeds, a signal in the exocarp and/or mesocarp, and a moderate signal in the endocarp, in line with observed expression in complete tomato fruits (Moxon et al., 2008). The U6 signal was also clearly observed in the seeds and a lower signal in the pericarp tissues. Notably, the nucleolar U6 signal may also change during development, as previously reported (e.g., Kou et al., 2012).

Subsequently, we repeated the miR164 detection in different fruit crops, now in strawberry (botanically speaking not a berry), guava, and again tomato, but for each two different developmental stages were used (**Figure 3**). Again, the strongest hybridization signal can be observed in seeds and a lower expression in pericarp tissues. Notably, a clear signal can be seen from the seeds that are on the outside in the case of strawberry (**Figure 3**).

Furthermore, we also tried floral buds to see if we could detect signal for miR164 and for miR159. We used transverse cuts of two developmental stages of floral buds from agave to make the tissue prints (**Figure 4**). We observed signal for both miRNAs, for miR164 mostly in placenta/transmitting tract tissue and for miR159 mostly in ovules.

The observed hybridization patterns in the different experiments for miR164, miR159, and nucleolar U6 are different, indicating that the observed signals are not background. Notably, when we used banana fruit for the tissue print, which left a lot of tissue behind on the membrane, we observed signal for the probe against miR164, but also for the sense miR164 negative control (Supplementary Figure S1). For the tissue prints of the other fruits no signal was observed with the miR164 sense probe (Supplementary Figure S2). So, caution should be taken with making conclusions when printing very soft tissues. However, we cannot exclude the possibility that miR164^∗^ in banana fruit is stable and detectable, as the detection of some other miRNA^∗^s has been reported (Zhang et al., 2011; Manavella et al., 2013). A possibility is to use another miRNA sense probe to distinguish between background and real signal.

### DETECTION OF miRNAs IN FRUIT JUICE BY DOT BLOT HYBRIDIZATION

**FIGURE 5:**
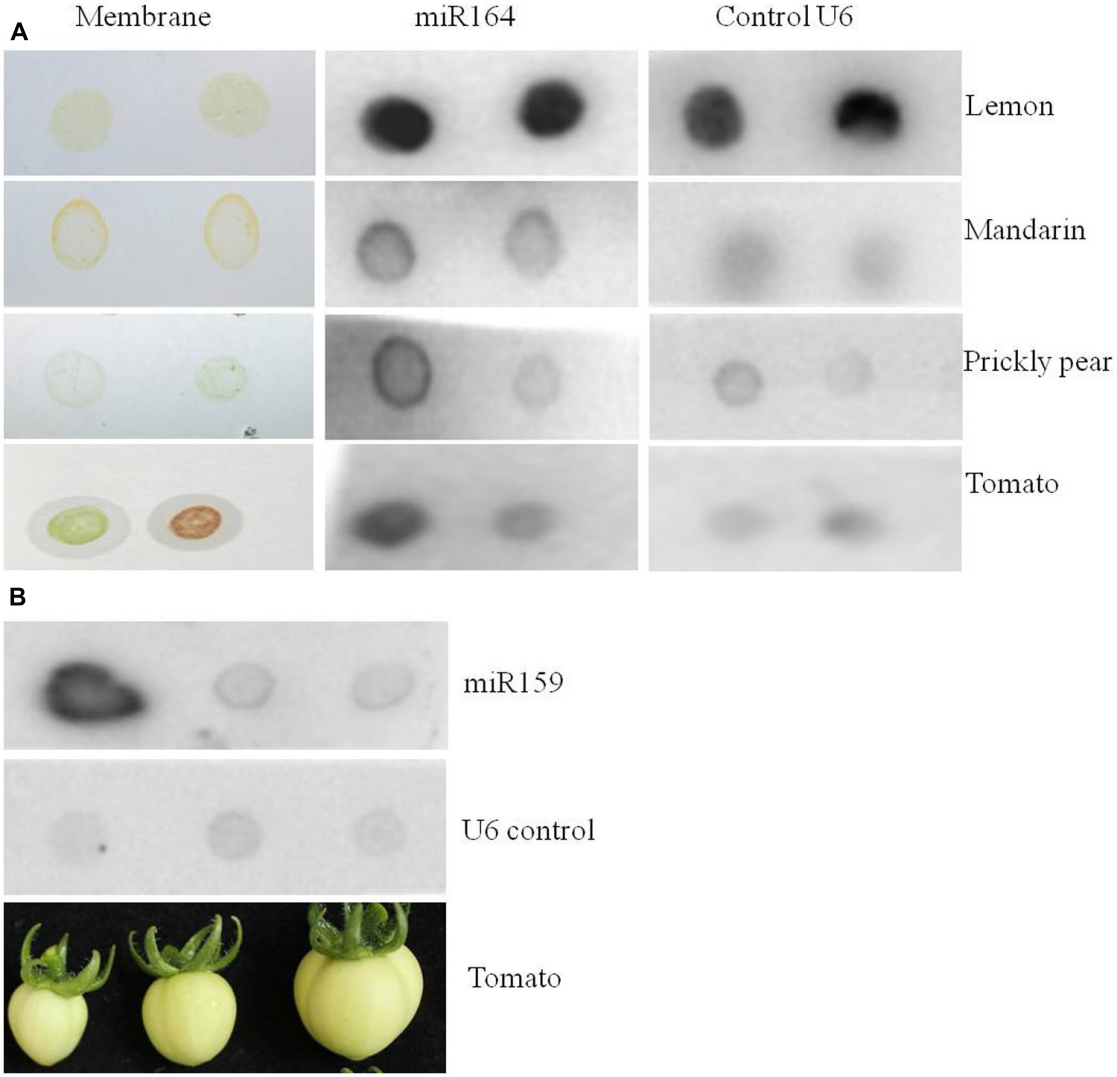
**miR164 and miR159 detection in fruit juices of different plant species by dot blot hybridization.** Five microliter of fruit juice were spotted on membranes and the miRNAs were detected by hybridization assays. **(A)** Expression of miR164 in lemon and mandarin juice, and in crude extract of green and ripe stages of prickly pear cactus and tomato fruits. **(B)** Expression of miR159 in different developmental stages of tomato crude extracts. The tomato fruit stages used for the crude extracts are shown below the membrane.

Sometimes it might not be important to know exactly where in the tissue the miRNA is expressed, but for instance just presence or absence, which could be useful to analyze genetically modified crops. We reasoned that it should be possible to detect miRNA expression in fruit juice (or crude extract). For this we prepared membranes with 5 μl fruit juice of lemon, mandarin, prickly pear cactus fruit, and tomato fruit. After applying the juice to the membrane, the membranes were fixed and then hybridized with the probe against miR164 (**Figure 5A**). Indeed, a clear signal was observed for miR164 as well as for the positive control U6. Furthermore, we tried fruit juice of three different developmental stages of tomato fruits and hybridized it with the probe against miR159 (**Figure 5B**). Also for miR159 a clear signal was observed, which was the highest in the youngest fruit stage, suggesting that miR159 is more expressed at that stage. In summary, this is an alternative way to quickly detect the expression of a miRNA.

## CONCLUSION

Here we provide a simple, rapid, and useful protocol to detect miRNAs from different tissues and organs of plant species. Using this tissue printing hybridization protocol we were able to determine the spatial–temporal expression pattern of miR159 and miR164 in different tissues. Moreover, we showed the possibility to detect the expression of miRNAs in fruit juice using a dot blot hybridization approach. This allows for instance the semi-high throughput screening for the presence of a miRNA in fruits when no tissue localization is required.

## AUTHOR CONTRIBUTIONS

FFRC did the major experimental work. REG contributed to the hybridization assays. FFRC, ACH, NMM, and SDF conceived the project and designed the experiments. FFRC and SDF drafted the manuscript. All authors read and approved the final manuscript.

## Conflict of Interest Statement

The authors declare that the research was conducted in the absence of any commercial or financial relationships that could be construed as a potential conflict of interest.

## References

[B1] Chavez Montes R. A. Rosas-Cárdenas F. F. De Paoli E. Accerbi M. Meyers B. C. Rymarquis L. A. (2014). Sample sequencing of vascular plants demonstrates widespread conservation and divergence of microRNAs. *Nat. Commun.* 5 3722 10.1038/ncomms4722 24759728

[B2] Chen C. Ridzon D. A. Broomer A. J. Zhou Z. Lee D. H. Nguyen J. T. (2005). Real-time quantification of microRNAs by stem-loop RT-PCR. *Nucleic Acids Res.* 33 e179. 10.1093/nar/gni178 PMC1292995 16314309

[B3] Esteves F. Teixeira Santos M. Eiras-Dias J. E. Fonseca F. (2013). Molecular data mining to improve antibody-based detection of Grapevine leafroll-associated virus 1 (GLRaV-1). *J. Virol. Methods* 194 258–270 10.1016/j.jviromet.2013.09.004 24056263

[B4] Holland N. Holland D. Helentjaris T. Dhugga K. Beatriz X.-C. Delmer D. P. (2000). A comparative analysis of the plant cellulose synthase (CesA) gene family. *Plant Physiol.* 123 1313–1323 10.1104/pp.123.4.1313 10938350 PMC59090

[B5] Jolie R. P. Duvetter T. Vandevenne E. Van Buggenhout S. Van Loey A. M. Hendrickx M. E. (2010). A pectin-methylesterase-inhibitor-based molecular probe for in situ detection of plant pectin methylesterase activity. *J. Agric. Food Chem.* 58 5449–5456 10.1021/jf100248u 20380375

[B6] Kidner C. Timmermans M. (2006). In situ hybridization as a tool to study the role of microRNAs in plant development. *Methods Mol. Biol.* 342 159–179 10.1385/1-59745-123-1:159 16957374

[B7] Kou S. J. Wu X. M. Liu Z. Liu Y. L. Xu Q. Guo W. W. (2012). Selection and validation of suitable reference genes for miRNA expression normalization by quantitative RT-PCR in citrus somatic embryogenic and adult tissues. *Plant Cell Rep.* 31 2151–2163 10.1007/s00299-012-1325-x 22865195

[B8] Manavella P. A. Koenig D. Rubio-Somoza I. Burbano H. A. Becker C. Weigel D. (2013). Tissue-specific silencing of *Arabidopsis* SU(VAR)3-9 HOMOLOG8 by miR171a. *Plant Physiol.* 161 805–812 10.1104/pp.112.207068 23204429 PMC3561020

[B9] Mochizuki T. Ohki S. T. (2015). Detection of plant virus in meristem by immunohistochemistry and in situ hybridization. *Methods Mol. Biol.* 1236 275–287 10.1007/978-1-4939-1743-3_20 25287510

[B10] Moxon S. Jing R. Szittya G. Schwach F. Rusholme Pilcher R. L. Moulton V. (2008). Deep sequencing of tomato short RNAs identifies microRNAs targeting genes involved in fruit ripening. *Genome Res.* 18 1602–1609 10.1101/gr.080127.108 18653800 PMC2556272

[B11] Pall G. S. Hamilton A. J. (2008). Improved northern blot method for enhanced detection of small RNA. *Nat. Protoc.* 3 1077–1084 10.1038/nprot.2008.67 18536652

[B12] Pluskota W. E. Bradford K. J. Nonogaki H. (2011). Tissue-printing methods for localization of RNA and proteins that control seed dormancy and germination. *Methods Mol. Biol.* 773 329–339 10.1007/978-1-61779-231-1_19 21898264

[B13] Qu L. Q. Tada Y. Takaiwa F. (2003). In situ western hybridization: a new, highly sensitive technique to detect foreign and endogenous protein distribution in rice seeds. *Plant Cell Rep.* 22 282–285 10.1007/s00299-003-0683-9 12937942

[B14] Rosas-Cárdenas F. F. Caballero-Pérez J. Ramos X. G. Cruz-Hernández A. Marsch-Martínez N. de Folter S. (2015). miRNA expression during prickly pear cactus fruit development. *Planta* 241 435–448 10.1007/s00425-014-2193-0 25366556

[B15] Rosas-Cárdenas F. F. Durán-Figueroa N. Vielle-Calzada J. P. Cruz-Hernández A. Marsch-Martínez N. de Folter S. (2011). A simple and efficient method for isolating small RNAs from different plant species. *Plant Methods* 7 4 10.1186/1746-4811-7-4 PMC3056851 21349188

[B16] van Rooij E. (2011). The art of microRNA research. *Circ. Res.* 108 219–234 10.1161/CIRCRESAHA.110.227496 21252150

[B17] Várallyay É. Havelda Z. (2011). Detection of microRNAs in plants by in situ hybridisation. *Methods Mol. Biol.* 732 9–23 10.1007/978-1-61779-083-6_2 21431702

[B18] Varner J. E. Ye Z. (1994). Tissue printing. *FASEB. J.* 8 378–384. 8168688 10.1096/fasebj.8.6.8168688

[B19] Voinnet O. (2009). Origin, biogenesis, and activity of plant microRNAs. *Cell* 136 669–687 10.1016/j.cell.2009.01.046 19239888

[B20] Yin J. Q. Zhao R. C. Morris K. V. (2008). Profiling microRNA expression with microarrays. *Trends Biotechnol.* 26 70–76 10.1016/j.tibtech.2007.11.007 18191262

[B21] Zhang X. Zhao H. Gao S. Wang W. C. Katiyar-Agarwal S. Huang H. D. (2011). *Arabidopsis* argonaute 2 regulates innate immunity via miRNA393(^∗^)-mediated silencing of a Golgi-localized SNARE gene, MEMB12. *Mol. Cell* 42 356–366 10.1016/j.molcel.2011.04.010 21549312 PMC3101262

