# Supplementary figures and images for: An efficient method for miRNA detection and localization in crop plants
Source: Front Plant Sci. 2015 Mar 3;6:99. doi: 10.3389/fpls.2015.00099 (PMC4347446; doi:10.3389/fpls.2015.00099)

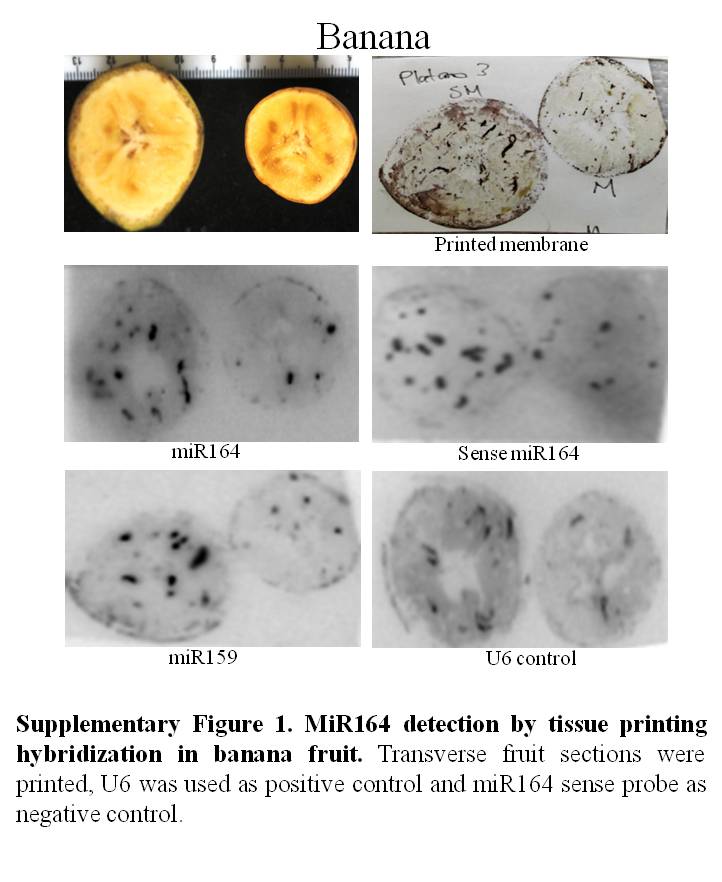

Supplement: Supplementary file 1 [file image_1.jpeg]

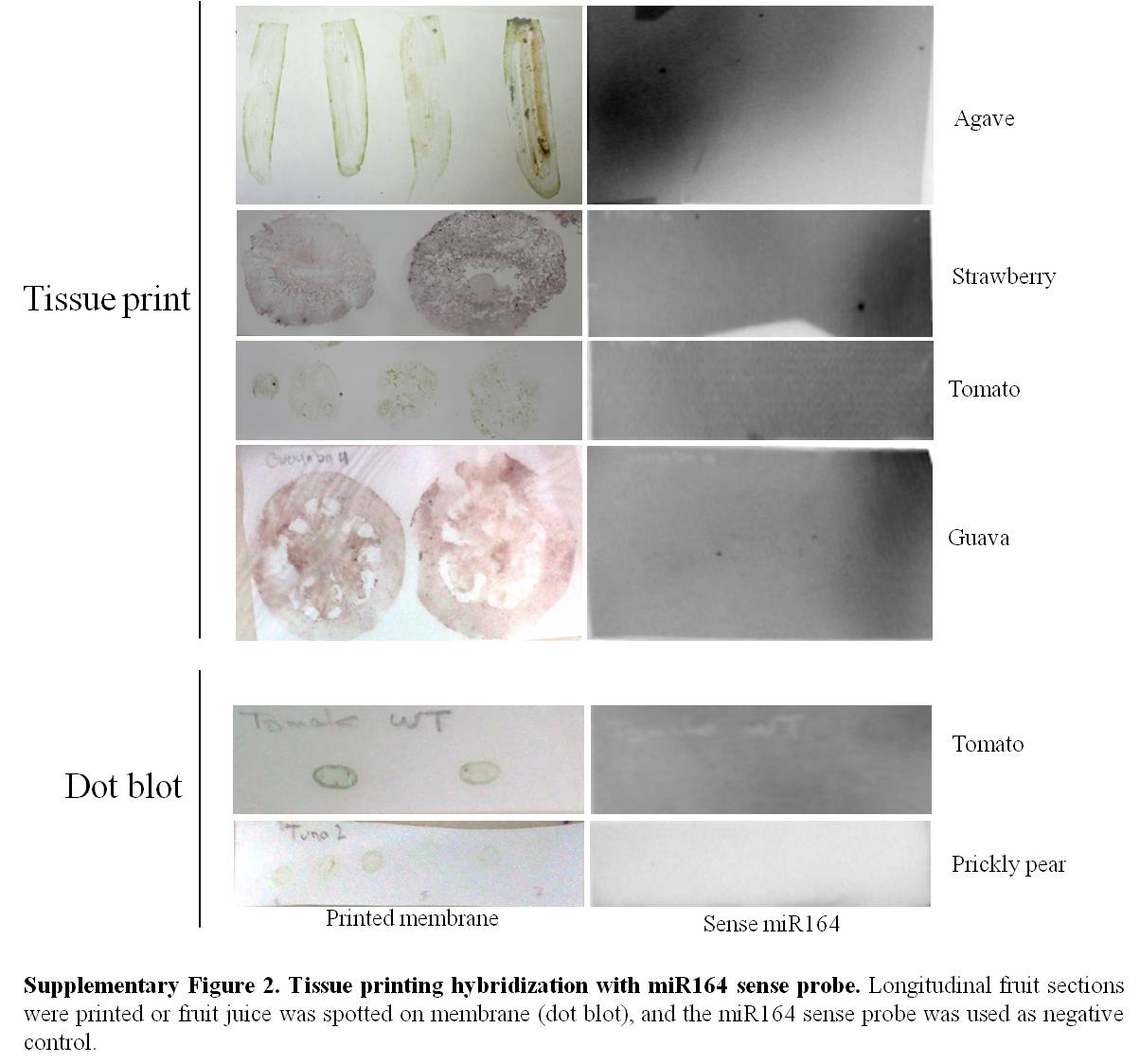

Supplement: Supplementary file 2 [file image_2.jpeg]
